# Caregiver burden reduction program among family caregivers of patients undergoing coronary artery bypass graft surgery: designing and evaluating

**DOI:** 10.1186/s12912-025-03918-9

**Published:** 2025-10-09

**Authors:** Fateme Beheshtaeen, Zahra Molazem, Majid Najafi Kalyani, Zinat Mohebbi

**Affiliations:** Department of Nursing, School of Nursing and Midwifery, Shiraz University of Medical Sciences, Shiraz, Iran

**Keywords:** Caregiver burden, Family caregivers, Coronary artery bypass graft

## Abstract

**Background:**

Caregiver burden, with its negative consequences for family caregivers of patients undergoing coronary artery bypass graft (CABG) surgery, can adversely affect the healthcare system. Given the widespread impact of caregiver burden, designing and implementing a program to reduce this burden within the context of available resources and cultural considerations is deemed essential. The aim of this study was to design, implement, and evaluate a program to reduce caregiver burden among family caregivers of patients undergoing CABG surgery.

**Methods:**

In this study, a caregiver burden reduction program was initially designed based on the Ewles and Simnett model. Then, the designed program was implemented in the form of a randomized clinical trial involving 59 family caregivers (divided into intervention and control groups). The implemented program was then evaluated using questionnaire es assessing caregiver burden, general health, and quality of life among adult caregivers.

**Results:**

The findings revealed that the mean caregiver burden score after the intervention was lower in the intervention group compared to the control group (10.60 ± 9.27 vs. 16.78 ± 14.86, *P* < 0.001). Additionally, the mean general health score (37.26 ± 7.63 vs. 40.42 ± 12.39, *P* < 0.001) and quality of life score (67.00 ± 12.02 vs. 45.13 ± 13.00, *P* < 0.001) improved in the intervention group compared to the control group. Within-group analyses indicated that caregiver burden, general health, and quality of life improved in both the intervention and control groups post-intervention (*P* < 0.001).

**Conclusion:**

This study demonstrated that the caregiver burden reduction program had positive effects on reducing caregiver burden, improving general health, and enhancing the quality of life among family caregivers of patients undergoing CABG surgery. Given the program’s effectiveness, healthcare providers and managers can utilize it to make decisions aimed at improving the quality of services for caregivers in healthcare settings.

**Trial registration:**

The study was registered in “irct.behdasht.gov.ir” a website and online database with the number 77592 (2024-06-29).

**Supplementary Information:**

The online version contains supplementary material available at 10.1186/s12912-025-03918-9.

## Background

Cardiovascular diseases are among the leading causes of premature mortality worldwide [1]. It is predicted that by 2030, cardiovascular diseases will cause approximately 23 million deaths annually [2]. Iran bears the highest burden of cardiovascular diseases in the Eastern Mediterranean region, with these conditions accounting for 46% of all deaths and 20–23% of the disease burden in the country [3].

CABG surgery is a potentially life-saving treatment for cardiac patients. Family caregivers play a critical role in the successful recovery of patients’ post-discharge [4]. Post-discharge care for CABG patients includes managing post-surgical pain, wound care, ensuring adequate sleep and rest, monitoring weight, adhering to dietary and exercise regimens, scheduling follow-up medical visits, making lifestyle changes, managing treatment, and monitoring for post-surgical complications, all of which are expected to be performed by family caregivers [5].

CABG surgery can have debilitating physical and psychological consequences for both patients and their caregivers [4]. Caregivers often feel unprepared to provide care and lack sufficient understanding and knowledge of the disease process and their role in supporting patients, leading to increased uncertainty and distress [6]. A systematic review highlighted the complexity of the caregiver role, including the need for significant coordination, increased family responsibilities, loss of roles, and identity changes. Caregivers also reported feelings of abandonment by healthcare teams, providing care without medical consultation or support, receiving inadequate verbal or written information in hospitals, and making decisions in critical situations without sufficient knowledge [7]. Although caregiving is associated with familial and spiritual rewards, research indicates that the intensity and scope of this role contribute to caregiver burden [8].

Caregiver burden is a complex concept encompassing physical, psychological, emotional, social, and economic dimensions, impacting caregivers’ health and quality of life [9]. The extensive responsibilities of caregivers are associated with numerous challenges, often leading to negative outcomes such as impaired mental and physical health [10]. Studies have shown that caregiver burden negatively affects the quality of life of caregivers of cardiac patients. Additionally, increased caregiver burden and reduced quality of life can lead to mental health disturbances among caregivers [11].

Supportive educational programs appear to be one of the most effective strategies for reducing caregiver burden and enhancing health promoting behaviors among family caregivers [12]. Well-structured caregiving programs can play a significant role in improving the quality of care [13]. The promotion of health extends far beyond health education and encompasses a broader spectrum of activities. Health promotion models provide a clear framework for integrating health and social care [14].

The Ewles and Simnett model is one such framework, aiming to encourage the adoption of health behaviors among communities, individuals, and groups. The Ewles and Simnett model is versatile and has been applied in various domains, including palliative care [15] and the reduction of moral distress in nursing [16]. As this model involves a comprehensive assessment and prioritization of caregivers’ needs, with interventions designed based on objectives and implemented according to available resources, it can be used to execute interventions more precisely [17]. One of the key approaches of the Ewles and Simnett model is facilitating behavioral change to foster the adoption of a healthy lifestyle among individuals [18]. Considering that one of the objectives of reducing the caregiving burden is to promote health-enhancing behaviors among caregivers [19], the Ewles and Simnett model can be utilized to design programs aimed at alleviating the caregiving burden.

Iran’s healthcare system faces challenges in providing follow-up care for patients’ post-discharge, resulting in numerous caregiving difficulties for family caregivers, including disrupted social and familial relationships, physical problems, high costs, and negative emotions [20, 21].

Considering cultural and value-based differences among families and the need to account for available resources in healthcare systems, a comprehensive program addressing various caregiving dimensions is necessary. In existing studies, most interventions have been conducted without considering the needs of caregivers and solely based on the researchers’ previous assumptions and findings from literature reviews [8, 22], and so far, no comprehensive and extensive intervention has been implemented to reduce the caregiving burden. Therefore, this study aimed to design, implement, and evaluate a caregiver burden reduction program among family caregivers of patients undergoing CABG surgery.

## Methods

### Study design

This study is part of a sequential exploratory mixed-methods research project conducted between February 2023 and September 2024.

### Adherence to mixed methods reporting guidelines

We rigorously adhered to the GRAMMS (Good Reporting of a Mixed Methods Study) guidelines to ensure comprehensive reporting of our mixed-methods research [23] (Table S1).

Since the knowledge obtained through quantitative studies is unable to fully explain the condition of caregiving burden, and most interventions in existing quantitative research have been based on the researchers’ experiences or literature rather than being tailored to the specific context and needs of caregivers, it became necessary to conduct a qualitative study to clarify the current situation regarding caregiving burden. Subsequently, based on the findings derived from participants’ experiences and considering the available cultural and value-based conditions in Iranian society, a program capable of addressing the various dimensions of caregiving could be designed and implemented.

To achieve this, the study utilized a sequential exploratory mixed-methods design, consisting of three distinct phases. The first phase employed a qualitative approach to explore and conceptualize caregiver burden based on the experiences of family caregivers of CABG patients and to identify caregivers’ needs, forming the foundation for developing the intervention program. In the second phase, themes derived from the qualitative data, combined with findings from a literature review, were incorporated into the caregiver burden reduction program. In the third phase, the designed program was implemented through a clinical trial, and its effects on caregivers were assessed by three outcomes: Caregiver Burden, General Health, and Caregiver Quality of Life.

### Setting and participants

#### Qualitative phase

Participants included family caregivers of patients admitted to the cardiac intensive care units of two hospitals affiliated with Shiraz University of Medical Sciences, six weeks post-discharge following CABG surgery. Semi-structured interviews were conducted with 16 purposefully selected family caregivers. Data was analyzed by Graneheim and Lundman content analysis. Further details of this phase are published in a separate article [24] (Table S2, S3, S4).

#### Quantitative phase

This study was conducted on 59 caregivers (29 in the intervention group, with one excluded due to the patient’s death, and 30 in the control group) between May 2024 and September 2024. The research setting included hospitals and cardiac surgery clinics affiliated with Shiraz University of Medical Sciences.

### Inclusion and exclusion criteria

#### Inclusion criteria

One of family caregivers of the patient (next of kin) who is primarily responsible for patient care [15, 25], aged over 18 years, able to read and write, capable of understanding and speaking Persian and expressing emotions and experiences [22, 25], no prior experience caring for a patient undergoing CABG surgery [26], caring for a patient who underwent CABG surgery and was discharged at least five days prior, possessing a smartphone and the ability to use it [27], and willingness to participate in the study.

#### Exclusion criteria

Diagnosed chronic or severe illness based on self-report, caring for other patients with severe physical or mental chronic conditions in the family, diagnosed mental health or cognitive issues, employment as healthcare provider, withdrawal from the study, or patient death during the study [27–29].

### Intervention development based on Ewles and Simnett model

The caregiver burden reduction program was designed using the seven-stage Ewles and Simnett model [30].

#### Stage 1: Identifying needs and priorities

In this stage, the needs and challenges of family caregivers of patients undergoing CABG surgery, identified in the qualitative phase, were determined. These needs were prioritized for program design with input from a panel of experts and stakeholders, including nine family caregivers and six specialists (two nursing faculty members, one psychologist, one psychiatric nurse, one clinical supervisor, and one nurse working in the cardiac surgery unit). Stakeholders and experts rated the identified needs based on their priority/importance in contributing to or affecting caregiver burden, considering feasibility, on a scale of 1 to 5. The average score for each need was calculated, and needs were prioritized based on these scores.

#### Stage 2: Setting aims and objectives

General and specific objectives for the program were formulated based on the prioritized needs.

#### Stage 3: Deciding the best way of achieving the aims

Strategies and program content were determined based on the qualitative findings from the first phase and a literature review on caregiver burden. The literature review was conducted without time limitation with relevant keywords (see the table S3 in supplementary file). The findings are presented in Table 1.

The best strategies were selected based on criteria such as higher acceptance among caregivers, ease of implementation, cost-effectiveness, user-friendliness, and effectiveness in achieving objectives.

**Table 1:** Findings from qualitative study and literature review

Strategies proposed from qualitative findings	Findings from literature review
• Stress management • Sleep improvement strategies • Self-care • Education on increasing participation and task delegation • Managing interpersonal relationships (caregiver-family) • Patient care • Coping strategies • Social communication improvement • Spiritual care strategies	• Educational strategies: Individual and group practical patient care training, provision of pamphlets/educational booklets, and use of audio-visual media (telephone, internet, applications). • Non-educational strategies: Supportive, psychological, spiritual, respite care, lifestyle changes, and complementary medicine

Considering feasibility, available resources, budget, caregiver and patient cooperation, and existing conditions, appropriate strategies were selected. The implementation method and timeline for interventions were then determined. A set of interventions aimed at reducing caregiver burden was designed (Tables 2 and 3). The content of the program was reviewed and revised by a panel of experts (two nursing faculty members, one cardiac surgeon, one clinical psychologist, one psychiatric nurse, and one clinical supervisor).

**Table 2:** Caregiver burden reduction program based on the Ewles and Simnett model

Identified Needs	General Objective	Interventions
- Psychological distress of caregivers - Reduced patience and tolerance of caregivers - Limited social interactions	Improving the psychological conditions and social relationships of caregivers to reduce caregiver burden in family caregivers	- Psychological interventions - Management of interpersonal relationships - Spiritual interventions
- Lack of caregiving knowledge - Learning difficulties among caregivers - Deficiencies in the educational program of healthcare staff	Enhancing caregiving knowledge to reduce caregiver burden in family caregivers	- Education on surgical care and post-operative complications - Receiving feedback from caregivers regarding the learning of educational materials in a Q&A format. - Providing educational programs in written and visual formats via virtual platforms
- Sleep and rest disturbances among caregivers - Physical problems of caregivers	Improving physical health and sleep/rest patterns of caregivers	- Sleep hygiene - Relaxing audio and video clips for nighttime - Psychological interventions - Self-care education for caregivers
- Increased household responsibilities - Weak family cooperation in caregiving - Intensified patient care duties - Excessive expectations from caregivers - Compulsion to accept caregiving role	Reducing processes causing role strain	- Counseling for caregivers on task delegation - Management of caregiver-family communication - Time management

**Table 3:** E-Health content provided in the caregiver burden reduction program

Topic	Content	Content Type	Delivery Time
Post-surgical care	- Overview of coronary artery bypass graft surgery - Care for surgical wounds and signs of infection - Healthy lifestyle (smoking cessation, diet, management of hypertension, diabetes, and hyperlipidemia) - Physical activity - Post-operative physiotherapy - Post-operative complications and related care - Common medications	- Booklet as a PDF file - Video clip on physiotherapy (6 min) - Video clip on pulse checking (1.5 min)	First day of intervention
Stress management	- Definition of distress and eustress - Avoiding negative thinking - Strategies to reduce stress - Strategies for organizing life - Self-care methods - Problem-focused and emotion-focused coping - Anger management	- Audio file (19 min) - Written video clip (PowerPoint file) - Motivational video clips (5–10 min)	Second day of intervention (motivational video clips sent weekly)
Sleep improvement strategies	- Importance of sleep for caregivers - Causes and consequences of sleep deprivation in caregivers - Sleep improvement strategies (improving daily sleep and rest schedule, reducing tobacco use, optimizing bedroom environment, stress reduction, pre-sleep diet, seeking help for nighttime caregiving)	- Written video clip (PowerPoint file) - Relaxing nighttime video clips (5–10 min)	Third day of intervention (video clips sent nightly)
Time management	- Definition of time management - Personal planning for self-care - Defining roles and responsibilities of others - Creating and prioritizing task lists - Learning to say no	Video clip (16 min)	Fourth day of intervention
Caregiver-patient interpersonal communication	- Impact of heart surgery on the patient’s physical and mental condition - Importance of effective caregiver-patient communication during recovery - Principles of effective communication	Audio file (12 min)	Fifth day of intervention
Caregiver-family and social interpersonal communication	- Caregiver roles during the caregiving period - Assertiveness skills - Strategies for dividing caregiving tasks within the family - Strategies for expressing emotions within the family - Conflict resolution strategies - Strategies for enhancing social interactions with others	Audio file (15 min)	Sixth day of intervention
Spiritual care	- Meaning in life - Gratitude - Mindfulness exercises - Hope and reliance on God - Strengthening patience during caregiving	- Audio file (13 min) - Two video clips (1–3 min)	Seventh day of intervention

#### Stage 4: Identifying resources

Required and available resources, additional resources, and necessary budget were identified. Professional expertise included the knowledge and skills of the research team, time required for program implementation, and necessary skills. Caregiver resources included free time, energy, and motivation. Influential individuals included patients and healthcare staff. Required facilities and equipment included smartphones, domestic and international messaging platforms, and internet access.

#### Stage 5: Plan evaluation methods

Quantitative evaluation methods were used to assess the program’s quantitative effects on reducing caregiver burden.

#### Stage 6: Set an action plan

All necessary and foreseeable activities required to implement the caregiver burden reduction program and achieve general and specific objectives were listed.

#### Stage 7: Action

The designed program was implemented in the form of a randomized clinical trial.

### Implementation method

After obtaining ethical approval from the Ethics Committee of Shiraz University of Medical Sciences, the researcher contacted caregivers of patients who had undergone CABG surgery and were discharged five days prior. The study’s objectives were fully explained to the caregivers, and written informed consent was obtained in person at the clinic for those willing to participate.

Based on the study by Moieni et al., with α = 0.05 and a statistical power (1-β = 0.99), a sample size of 27 individuals per group was determined [8]. Accounting for a 10% dropout rate, the final sample size was set at 30 individuals per group. The correlation between the total Caregiver Burden Inventory (CBI) scores before and after the intervention was assumed to be 0.50, yielding an effect size of 0.869.

$$\:\varvec{n}=\frac{{({\varvec{t}}_{\varvec{n}-1,\raisebox{1ex}{$\varvec{\alpha\:}$}\!\left/\:\!\raisebox{-1ex}{$2$}\right.}+{\varvec{t}}_{\varvec{n}-1,\varvec{\beta}})}^{2}\:\left({\varvec{\sigma\:}}_{\varvec{d}}^{2}\right)\:}{{({\varvec{\mu\:}}_{1}-{\varvec{\mu\:}}_{2})}^{2}}$$

$$\text{S}_1=14.3,\:\text{S}_2=10.0,\:\rm \mu_1= 30.08,\:\rm \mu_2= 19.2$$

Participants were divided into intervention and control groups (30 in each). Random allocation was performed using block randomization, with a web-based random block list generator creating 15 blocks of four. Both groups received routine care and follow-up from the cardiac surgery intensive care unit, with the control group receiving one follow-up phone call by the researcher. The intervention group received additional interventions to reduce caregiver burden from day five to six weeks post-discharge, as this is the typical recovery period for CABG patients to resume daily activities [22].

Part of the intervention program was delivered electronically, with additional interventions provided through follow-up. The electronic program consisted of eHealth content sent via social media, including audio, video, and written materials addressing patient care for CABG patients, stress management, sleep improvement strategies, time management, interpersonal relationships (caregiver-patient and caregiver-family), and spiritual care. These materials, each lasting 10–20 min, were sent daily for one week.

From weeks two to five, interventions were conducted through follow-up with review of the contents. The researcher followed up with caregivers via phone calls and social media, addressing their questions, ambiguities, and educational and psychological needs individually. Caregivers were provided with a time diary, including 16 self-care recommendations, which they completed daily and sent weekly to the researcher via social media. These recommendations included prayers and gratitude, stress management strategies, appropriate diet, sleep improvement strategies, communication with patients and family, and relaxing activities.

To ensure learning of caregiving education, multiple-choice questions were prepared, and caregivers were asked to answer them at the end of the first week of intervention. If caregivers reported patient needs, individual telephone consultations were provided to promote treatment adherence and self-care. These mechanisms helped ensure that participants actively engaged with the intervention materials. Five weeks post-intervention, both groups completed the study questionnaires assessing caregiver burden, general health, and quality of life.

### Measurement tools

Four questionnaires were used: Demographic Information, Caregiver Burden Inventory (CBI), General Health Questionnaire (GHQ), and Adult Carer Quality of Life Questionnaire (AC-QoL).

- **Demographic Information Questionnaire**: Included age, gender, education, occupation, marital status, income level, use of government assistance, financial dependency, and access to substitute caregivers, designed based on literature review and expert opinions (Table S6).
- **Caregiver Burden Inventory (CBI)**: Measures five dimensions of caregiver burden: time-dependent (5 items), developmental (5 items), physical (4 items), social (5 items), and emotional (5 items). It consists of 24 questions on a 5-point Likert scale (strongly disagree to strongly agree), with higher scores indicating greater burden [31]. The Persian version’s validity and reliability were assessed by Shafiezadeh et al. (2019) in caregivers of Alzheimer’s patients. Construct validity was evaluated through known-group comparisons, and convergent validity was assessed via correlations with the Beck Depression and Anxiety Inventories. Internal consistency (Cronbach’s alpha) was 0.93, and test-retest reliability over two weeks was 0.96 [32].
- **General Health Questionnaire (GHQ-28)**: Designed by Goldberg (1997), it includes 28 questions across four subscales: somatic symptoms (7 questions), anxiety and insomnia (7 questions), social dysfunction (7 questions), and severe depression (7 questions). Scores range from 0 to 84, with higher scores indicating poorer general health [33]. The Persian version’s psychometric properties were evaluated by Ebrahimi et al. (2007) in healthy individuals and psychiatric patients. Exploratory factor analysis showed that the dimensions explained 69.55% of the total variance. Criterion and diagnostic validity showed a correlation of 0.781, (*P* < 0.001). Cronbach’s alpha was 0.97, indicating high reliability [34].
- **Adult Carer Quality of Life Questionnaire (AC-QoL)**: Measures eight dimensions: caregiving support (5 items), caregiving choice (5 items), caregiving stress (5 items), financial matters (5 items), personal growth (5 items), sense of value (5 items), caregiving ability (5 items), and carer satisfaction (5 items). It uses a 4-point Likert scale (never, sometimes, often, always), with scores ranging from 0 to 120. Higher scores indicate better quality of life [35]. The Persian version, validated by Rouhi Safaei et al. (2022) in stroke caregivers, has seven dimensions after excluding caregiving stress. After removing six items, the questionnaire was reduced to 34 items. Exploratory factor analysis extracted seven factors explaining 41.04% of the variance. Total internal consistency was 0.899, with Cronbach’s alpha and McDonald’s omega above 0.7 for all dimensions. Confirmatory factor analysis indicated moderate model fit [36].

### Data analysis

Based on the Shapiro wilk results, parametric tests (independent t-test) were used for normally distributed variables, and non-parametric equivalents (Mann-Whitney and Wilcoxon tests) were used for non-normally distributed variables. The significance level was set at 0.05. Descriptive and inferential statistical tests were used for data analysis.

## Results

Demographic characteristics of the intervention and control groups are presented in Table 4. Chi-square and Fisher’s exact tests showed no significant differences between the groups in terms of gender, marital status, education, occupation, government financial assistance, financial independence, or access to substitute caregivers (*P* ≥ 0.05).

**Table 4:** Demographic characteristics of participants

Variable	Category	Intervention Group *N* (%)	Control Group *N* (%)	*P*-value
Gender	Female	26 (49.1%)	27 (50.9%)	0.648
	Male	3 (50%)	3 (50%)	
Marital Status	Single	8 (61.5%)	5 (38.5%)	0.673
	Married	20 (45.5%)	24 (54.5%)	
	Widowed	1 (50%)	1 (50%)	
Education	Below High School	7 (63.6%)	4 (36.4%)	0.427
	High School	11 (47.8%)	12 (52.2%)	
	Associate Degree	1 (25%)	3 (75%)	
	Bachelor’s or Higher	10 (42.1%)	11 (57.9%)	
Occupation	Self-Employed	3 (60%)	2 (40%)	0.197
	Employee	8 (57.1%)	6 (42.9%)	
	Unemployed/Homemaker	18 (45%)	22 (55%)	
Government Financial Aid	Yes	6 (54.5%)	5 (45.5%)	0.748
	No	23 (47.9%)	25 (52.1%)	
Financial Independence	Dependent	18 (48.6%)	19 (51.4%)	0.92
	Independent	11 (50%)	11 (50%)	
Access to Alternative Caregiver	Yes	15 (50%)	15 (50%)	0.895
	No	14 (48.3%)	15 (51.7%)	

Between-group comparisons of mean and standard deviation of caregiver burden scores showed no significant difference before the intervention (*P* = 0.634). Post-intervention, the mean caregiver burden score was 10.60 ± 9.27 in the intervention group and 16.78 ± 14.86 in the control group. The Mann-Whitney test indicated a significant difference post-intervention (*P* < 0.001). Within-group Wilcoxon tests showed significant differences in caregiver burden scores before and after the intervention in both the intervention (*P* < 0.001) and control groups (*P* = 0.001) (Table 5).

**Table 5:** Between- and within-group comparison of caregiver burden mean scores before and after intervention

	Intervention Group Mean (SD) Med (IQR)	Control Group Mean (SD) Med (IQR)	Between-Group *P*-value
Before Intervention	86.34 (10.74)	84.93 (11.85)	0.634 ^a^
After Intervention	60.10 (9.27) 60. 00 (12.5)	78.16 (14.86) 75.00 (29.5)	< 0.001 ^b^
Within-Group P-value	< 0.001	0.001	

a Independent T test, b Mann-whitney

Between-group comparisons of mean and standard deviation of general health scores showed no significant difference before the intervention (*P* = 0.562). Post-intervention, a significant difference was observed (*P* < 0.001). Within-group comparison shows significant differences in general health scores in the intervention group (*P* < 0.001) and control group (*P* = 0.042) (Table 6).

**Table 6:** Between- and within-group comparison of mean scores of general health before and after intervention

	Intervention Group Mean (SD)	Control Group Mean (SD)	Between-Group *P*-value
Before Intervention	47.79 (11.06)	46.1 (10.37)	0.562 ^a^
After Intervention	26.37 (7.63)	42.4 (12.39)	< 0.001^a^
Within-Group P-value	< 0.001	0.042	

a Independent t test

Independent t-tests showed no significant difference in mean quality of life scores before the intervention (*P* = 0.434). Post-intervention, a significant difference was observed (*P* < 0.001). Statistical tests indicated significant within-group differences in quality of life scores in both the intervention (*P* < 0.001) and control groups (*P* = 0.002) (Table 7).

**Table 7:** Between- and within-group comparison of quality of life mean scores before and after intervention

	Intervention Group Mean (SD)	Control Group Mean (SD)	Between-Group *P*-value
Before Intervention	36.95 (15.50)	39.76 (11.55)	0.434 ^a^
After Intervention	67.00 (12.02)	45.13 (13.00)	< 0.001 ^a^
Within-Group P-value	0.000	0.002	

a Independent t test

As mean scores for caregiver burden, general health, and quality of life were significant in both groups post-intervention (*P* < 0.001), independent t-tests were used to compare the mean differences in scores before and after the intervention, showing a significant difference (*P* < 0.001). This indicates that the caregiver burden reduction program had a greater impact on reducing caregiver burden, improving general health, and enhancing quality of life in the intervention group compared to the control group (Table 8).

**Table 8:** Between-Group comparison of mean difference in care Burden, general Health, and quality of life scores before and after intervention

Variable	Intervention Group Mean (SD)	Control Group Mean (SD)	*P*-value
Care Burden Difference	-26.24 (8.74)	-6.76 (9.56)	< 0.001^a^
General Health Difference	21.41 (7.05)	3.76 (8.77)	< 0.001^a^
Quality of Life Difference	30.03 (7.88)	5.36 (8.18)	< 0.001^a^

a Independent t test

## Discussion

This study evaluated the impact of a caregiver burden reduction program based on the Ewles and Simnett model on caregiver burden, general health, and quality of life among family caregivers of patients undergoing CABG surgery. Given that the Ewless and Simmnet model can promote health through various approaches, this study employed two specific strategies—educational interventions and behavioral change—to reduce the caregiving burden among caregivers of CABG patients.

The results showed a significant difference in mean caregiver burden, general health and quality of life scores post-intervention between the intervention and control groups, indicating the program’s effectiveness in reducing caregiver burden and improving general health and quality of life in the intervention group.

Since psychological, physical, social, economic, and emotional problems of caregivers during the caregiving period contribute to the caregiving burden [37], addressing these elements can help reduce the caregiving burden. On the other hand, it has been shown that general health, which includes somatic symptoms, anxiety and insomnia, social dysfunction, and depression, is a predictor of the caregiving burden in family caregivers [38].

The various components of the caregiver burden reduction program could help to alleviate depression, social isolation, and emotional exhaustion commonly experienced by caregivers.

Caregivers of cardiac surgery patients often face high stress levels due to the complexity of care required and the emotional burden of the patient’s health status. Stress management training, for instance, may have directly countered emotional fatigue, consistent with previous studies highlighting its effectiveness in reducing caregiver stress and anxiety [39]. Adopting sleep hygiene practices can improve both mental and physical well-being, thereby lessening the indirect perception of burden [40]. Since stress and psychological manifestations can exacerbate caregiving demands by disrupting sleep and quality of life, it seems that sleep-enhancing interventions can reduce caregiving burden by improving quality of life [41]. In this study, the majority of caregivers had an educational level below a high school diploma, which may indicate a limited ability to access up-to-date information regarding the care of cardiac surgery patients. As inadequate knowledge can contribute to the caregiving burden [24], enhancing caregivers’ knowledge has proven effective in reducing this burden. In this regards, educational interventions that provide detailed information about the surgical procedure, postoperative care, potential complications, and rehabilitation processes help to reduce caregivers’ anxiety and uncertainty.

On the other hand, in the current program, the strategies for promoting social support such as resolving conflicts of caregivers with families and patients, as well as the division of caregiving responsibilities among family members, and effective communication had led to a reduction in their psychological tensions and physical fatigue, which in turn reduced the caregiving burden and improved the general health of the caregivers. Diaz et al. (2019) show that social support mediated the relationship between perceived burden and general health [42]. Time management training was another strategy in the present program which provides caregivers with tools to optimize task organization, helping to alleviate the strain of managing multiple responsibilities.

Spiritual interventions, such as logotherapy and mindfulness practices, were also employed in this program to mitigate the caregiving burden. Spiritual well-being is frequently linked to fostering a sense of support, purpose, and effective coping strategies during difficult periods [43]. Caregivers, who often encounter significant stress and responsibilities, may find that a strong spiritual foundation aids in managing their duties and deriving meaning from their roles. Research has underscored the importance of spiritual health as a vital resource for caregivers, enabling them to address the emotional and psychological demands of their work. Additionally, spiritual well-being can enhance resilience and cultivate hope among caregivers [44].

Studies by Dalirirad et al. (2021) [22] and Moieni et al. (2014) [8], which examined the impact of psycho-educational interventions on caregiver burden, reported similar reductions in caregiver burden in the intervention group, consistent with the present study. However, those studies delivered interventions during patient hospitalization, before caregivers experienced caregiving at home. In contrast, the interventions in the present study were designed and implemented based on caregivers’ needs and priorities from the qualitative phase and literature review, offering greater comprehensiveness. Additionally, to ensure the accuracy of baseline data, the fifth day post-discharge was designated as the data collection point, a practice not adhered to in previous studies.

In Iran, studies examining the impact of supportive psycho-educational interventions on anxiety among caregivers of cardiac surgery patients have reported results consistent with this study [26]. The findings of other studies support the relationship between caregiver burden and impaired quality of life, with psychological distress acting as a mediator [9, 45]. Consistent with the findings of this study, Cassidy et al. (2021) demonstrated that a combination of supportive measures, such as telephone support, education, and written resources, led to reduced depression and improved quality of life among caregivers of cardiac patients [46].

A study by Ågren et al. (2015) evaluating a multidisciplinary supportive program for caregivers of CABG patients over one year found no significant difference in quality of life between groups, though the intervention group showed improvement compared to baseline. Depression data also showed no significant difference, contrasting with this study’s findings [47]. This discrepancy may be due to differences in data collection timing, intervention duration, and cultural context.

In addition to eHealth interventions, this study included telephone follow-ups and opportunities for caregivers to consult freely with researchers via social media. Communication technologies, such as telephones, computers, and smartphones, provide cost-effective methods for education and consultation, eliminating distance barriers and improving access for caregivers and patients [48]. A study by Namjoo et al. (2021) on telenursing’s impact on caregiver burden in heart failure patients showed a significant reduction in caregiver burden in the intervention group, consistent with this study [48]. However, a meta-analysis by Li et al. (2022) found that e-Health interventions improved depression and quality of life in cancer caregivers but had no effect on caregiver burden [49].

Within-group comparisons showed that caregiver burden decreased, and general health and quality of life improved in both groups post-intervention. To confirm the program’s effectiveness, mean score differences were analyzed, showing a greater impact in the intervention group. It should be mentioned that caregiving for CABG patients differs from chronic conditions like dementia or cancer, as it is typically a shorter-term role, ending 2–3 months post-surgery [50]. Thus, the reduction in the caregiving burden observed in the control group may be attributed to the fact that patients without severe post-operative complications typically recover more quickly. Consequently, as patients improve over time, the negative impacts of caregiving diminish, resulting in a lighter caregiving burden for caregivers.

This study was conducted over a five-week period, which is relatively short. Additionally, the lack of a long-term follow-up limits the ability to assess the sustained impact of the intervention. Since evaluating outcomes over longer intervals provides a more comprehensive understanding of the caregiving burden, it is recommended that future studies incorporate longer-term interventions with extended follow-up periods to better elucidate the trajectory of the caregiving burden in this context.

### Strengths and limitations

This study is the first to design a caregiver burden reduction program for CABG patients using qualitative findings from caregivers’ experiences, a literature review, and expert opinions within a randomized clinical trial with a control group to minimize bias. Virtual and individualized interventions minimized the need for in-person hospital visits, saving time, energy, and costs.

This study has several limitations, the most notable being the absence of an extended follow-up period to assess the lasting impact of the intervention. Variations in participants’ engagement levels with social media-based content may exist. Additionally, the relatively small sample size may limit the generalizability of the findings, particularly to diverse caregiving populations. Future studies should prioritize longer follow-up periods, larger and more diverse participant samples, and examine a broader range of caregiving environments, such as acute and chronic conditions, dementia care, and post-ICU care. Moreover, employing longitudinal mixed-method designs could yield valuable data on the long-term effects and contextual variations of these interventions.

### Implications for practice

This study has practical implications for healthcare providers, policymakers, and researchers. First, the findings demonstrate that caregiver burden can be effectively reduced by implementing a structured, needs-based intervention delivered through virtual platforms. This offers a scalable and cost-effective solution, especially in resource-constrained settings.

For healthcare systems, integrating such caregiver support programs into discharge planning and follow-up care protocols can improve both caregiver and patient outcomes. Nursing managers and health administrators may consider developing mobile-based caregiver education modules and virtual counseling services as part of post-operative care. From a policy perspective, the results support the inclusion of caregiver support in cardiac rehabilitation programs and health promotion strategies, especially for high-risk surgeries like CABG. Regarding future research, longitudinal studies with larger sample sizes are needed to assess long-term impacts and sustainability of such interventions. Exploring how demographic and cultural factors influence intervention effectiveness could further refine caregiver support models.

## Conclusion

This study demonstrated the positive effects of a caregiver burden reduction program on caregiver burden, general health, and quality of life. Given its practicality, healthcare providers and managers can use this program to make decisions aimed at improving quality of care for caregivers in healthcare settings.

## Supplementary Information

Below is the link to the electronic supplementary material.

Supplementary Material 1

## Data Availability

The datasets used and/or analyzed during the current study are available from the corresponding author on reasonable request.

## Abbreviations

AC-QoL: Adult Carer Quality of Life Questionnaire
CABG: Coronary Artery Bypass Graft
CBI: Caregiver Burden Inventory
GHQ: General Health Questionnaire
GRAMMS: Good Reporting of a Mixed Methods Study
Q&A: Question and answer
