# Supplementary material for: Caregiver burden reduction program among family caregivers of patients undergoing coronary artery bypass graft surgery: designing and evaluating
Source: BMC Nurs. 2025 Oct 9;24:1258. doi: 10.1186/s12912-025-03918-9 (PMC12512962; doi:10.1186/s12912-025-03918-9)
Supplement: Supplementary file 1 — Supplementary Material 1 [file 12912_2025_3918_MOESM1_ESM.docx]

**Table S1:** GRAMMS (Good Reporting of a Mixed Methods Study) guidelines

| Where in Manuscript | Reporting Item |
| --- | --- |
| Methods  Adherence to Mixed Methods Reporting Guidelines  Since the knowledge obtained through quantitative studies is unable to fully explain the condition of caregiving burden, and most interventions in existing quantitative research have been based on the researchers’ experiences or literature rather than being tailored to the specific context and needs of caregivers, it became necessary to conduct a qualitative study to clarify the current situation regarding caregiving burden. Subsequently, based on the findings derived from participants’ experiences and considering the available cultural and value-based conditions in Iranian society, a program capable of addressing the various dimensions of caregiving could be designed and implemented. | Describe the justification for using a mixed methods approach to the research question |
| This study utilized a sequential exploratory mixed-methods design, consisting of three distinct phases. The first phase employed a qualitative approach to explore and conceptualize caregiver burden based on the experiences of family caregivers of CABG patients and to identify caregivers’ needs, forming the foundation for developing the intervention program. In the second phase, themes derived from the qualitative data, combined with findings from a literature review, were incorporated into the caregiver burden reduction program. In the third phase, the designed program was implemented through a clinical trial, and its effects on caregivers were assessed by three outcomes: Caregiver Burden, General Health, and Caregiver Quality of Life. | Describe the design in terms of the purpose, priority and sequence of methods |
| **Methods (page 4-5)**  **Qualitative Phase**  Participants included family caregivers of patients admitted to the cardiac intensive care units of two hospitals affiliated with Shiraz University of Medical Sciences, six weeks post-discharge following CABG surgery. Semi-structured interviews were conducted with 16 purposefully selected family caregivers. Data was analyzed by Graneheim and Lundman content analysis. Further details of this  **Quantitative Phase**  This study was conducted on 59 caregivers (29 in the intervention group, with one excluded due to the patient’s death, and 30 in the control group) between May 2024 and September 2024. The research setting included hospitals and cardiac surgery clinics affiliated with Shiraz University of Medical Sciences.  **Implementation Method (page 9-12)** | Describe each method in terms of sampling, data collection and analysis |
| In the second phase, themes derived from the qualitative data, combined with findings from a literature review, were incorporated into the caregiver burden reduction program. In the third phase, the designed program was implemented through a clinical trial, and its effects on caregivers were assessed by three outcomes | Describe where integration has occurred, how it has occurred and who has participated in it |
| Not applicable | Describe any limitation of one method associated with the present of the other method |
| Not applicable | Describe any insights gained from mixing or integrating methods |

**Table S2: Explanation of the factors leading to the care burden of family caregivers of CABG patients**

| **Main Category** | **Subcategory** | **Sub-subcategory** |
| --- | --- | --- |
| Onslaught of physical and psychological tensions | Psychological reactions | Mental disturbances  Reduced resilience |
|  | Physical complications | Physical pain  Disruption of sleep and rest patterns |
| Confrontation with multiple roles and tasks | Role strain | Increased household duties  Intensification of care duties  Excessive expectations from the caregiver  The compulsion to accept care |
|  | Role conflict | Disruption in individual life  Job-related problems  Limitation of family/social relationships |
| Lack of resources | Information poverty | Lack of caring knowledge  Seeking for care information  Caregiver training requirements  Patient and caregiver learning challenges |
|  | Inefficient involvement of the family in the provision of care | The family's ineffective collaboration in care  Family interference in care  Ingratitude of family members towards the caregiver performance |
|  | Financial constraints | Exorbitant expenses  Decreased income  Consequences of financial poverty |
|  | Inefficient health system services | Lack of hospital facilities  Poor performance of HCPs  Poor access to HCP after discharge  Lack of proper training programs  Lack of access to a psychologist |

**Table S3: Explanation of the factors that reduce the care burden of family caregivers of CABG patients**

| **Main Category** | **Subcategory** | **Sub-subcategory** |
| --- | --- | --- |
| Adaptive coping | Problem-based coping | Physical self-care  Help-seeking  attempting to accelerate patient's recovery |
|  | Emotion-based coping | Sense of commitment and forgiveness  Striving to boost personal morale  Resorting to spirituality |
| Support system | Family and colleagues' support | Family involvement in care  Providing emotional support  Financial support  Support from colleagues |
|  | Patient advocacy | Trying to become independent  Gratitude for the caregiver  Patience and pain tolerance |
|  | Effective interactions of the HCPs | Appropriate response  Emotional support  Providing information support |

**Table S4: Some quotations for the first part of the study (qualitative study)**

| Category | quotations |
| --- | --- |
| Onslaught of physical and psychological tension | In relation to the stressfulness of care, one participant remarked: "The care period was very stressful for me. I was always stressed that something unfortunate could happen to my father after open heart surgery." P1 |
| Confrontation with multiple roles and tasks | One participant stated about her career issues and life disruption:  "Because of caring, my life is completely messed up now….My husband is so angry. Also, I'm afraid I'll lose my job, because of my absence from work." P2. |
| Lack of resources | One caregiver said about the ineffective collaboration of family members: "We are currently five siblings; however, all of my mother's caregiving responsibilities fall solely on me. My brothers and their wives do not help out at all. I am frustrated and on the verge of breaking down." P16  One of the participants stated about information poverty: "At the time of discharge, we were not provided with instructions on drug administration. I didn't know what this drug was used for. So I needed to obtain the drug details from the internet." P12 |
| Support systems | One participant explained regarding family and patient support: "At night, my two sons or my brother-in-law would come in shifts and take care of my husband, so that I wouldn't get tired. At night I was resting, furthermore, my husband was very patient. He did much of his work alone." P7 |
| Adaptive coping | One caregiver commented on her sense of commitment and dedication: "Even though I have back pain myself, I loved my brother and endured the pain. I was responsible for looking after him. I am quite pleased with what I did, I adore it." P9 |

**Table S5: Keyword used for literature review**

| Caregiver" OR "Carer(s)" OR "Care Giver" OR "Spouse Caregivers" OR "Family Caregivers" OR "Informal Caregivers" |
| --- |
| "AND |
| "Caregiver Burden" OR "Care Burden" OR "Care Giving Burden" OR "Caregiver Burnout" OR "Caregiver Exhaustion" OR "Caregiver Strain" |
| AND |
| “Health Planning” OR “Nursing Education” OR “Intervention” OR “Strategy” OR “Randomized clinical trial” OR “Clinical trial” OR “Trial” |

**Table S6: Demographic information questionnaire**

| 1 | Gender of caregiver: Male…... Female….... |
| --- | --- |
| 2 | Marital status: Single…... Married…... Divorced…... Separated…... Deceased....... |
| 3 | Level of education: Diploma…... Below diploma….. Associate degree.....  Bachelor’s degree..... Master’s degree and above...... |
| 4 | Age: …. |
| 5 | Occupation: Self-employed..... Worker…... Employee…... Unemployed....... Homemaker....... |
| 6 | Income: ....... million Tomans per month |
| 7 | Use of government assistance: Yes…... No....... |
| 8 | Financial dependency: Dependent....... Independent....... |
| 9 | Access to substitute caregiver: Yes....... No........ |
